# Physical performance reflects cognitive function, fall risk, and quality of life in community-dwelling older people

**DOI:** 10.1038/s41598-019-48793-y

**Published:** 2019-08-22

**Authors:** Shota Ikegami, Jun Takahashi, Masashi Uehara, Ryosuke Tokida, Hikaru Nishimura, Ayaka Sakai, Hiroyuki Kato

**Affiliations:** 1 Department of Orthopaedic Surgery, Shinshu University School of Medicine, 3-1-1 Asahi, Matsumoto, Nagano 390-8621 Japan; 2 Rehabilitation Center, Shinshu University Hospital, 3-1-1 Asahi, Matsumoto, Nagano 390-8621 Japan

**Keywords:** Epidemiology, Quality of life, Epidemiology

## Abstract

This report searched for relationships between physical performance and other health indices through a detailed investigation of a randomly sampled cohort from a basic town resident registry. Residents between the age of 50 and 89 years were randomly sampled from the basic resident registry of a cooperating town for construction of a 415-participant cohort that minimized selection bias. Cognitive function measures, annual fall frequency, and SF-8 as an HRQOL measure were the outcomes of interest. The impact of physical function on outcomes was predicted using multivariate regression models with age and gender as covariates. Knee muscle strength, grip strength, one-leg standing time, and two-step test score had a significant impact on cognitive scores and SF-8 physical component summary scores. A shift of -1 standard deviation for grip strength, the stand-up test, and the two-step test increased fall risk by 39%, 23%, and 38%, respectively. In conclusion, diminished physical performance is related to serious problems in older individuals, specifically cognitive deterioration, increased fall risk, and inability to maintain HRQOL. These factors are independent of age and gender. Thus, the higher physical function can be maintained in older people, the better the other conditions appear to remain.

## Introduction

As the proportion of older people is increasing worldwide, senior health is becoming an increasingly important health issue^1^. Typical changes accompanying advanced age include deteriorations in physical performance and cognitive function that can reduce living autonomy. In particular, falls are a very serious problem, often resulting in marked restriction of activities of daily living and health-related quality of life (HRQOL). The fall rate in the general older population is 28–35%^2,3^ and is even higher in those with low physical performance^4–7^.

Any level of sarcopenia or other motor dysfunction and accompanying falls represent important problems for institution-dwelling older people^8^, and decreases in muscular strength of the lower limbs and the deterioration of balance lead to falls even in the general population^9,10^. Cognitive impairment is also a threat to senior HRQOL and is simultaneously believed to lead to falls^11,12^. However, other than those, there are currently few high-quality reports investigating how physical performance impacts cognitive function, falls, and HRQOL in the community-dwelling senior population. We therefore randomly sampled residents between the age of 50 and 89 years from the basic resident registry of a cooperating town to construct a 415-participant cohort, termed the Obuse study cohort, that minimized selection bias.

This report searched for relationships between physical performance and other health indices through a detailed investigation of the Obuse study cohort to clarify the impact of physical performance on various problems threatening the HRQOL of the general older population.

## Results

Table 1 summarizes the results of the physical performance tests and other assessment items in this study. Regarding physical performance, males achieved significantly higher results than females for all parameters apart from one-leg standing. The differences between the genders for the stand-up test and two-step test were also statistically significant but smaller than those for muscle strength. Cognitive function according to the Montreal Cognitive Assessment (MoCA) and the Mini-Mental State Examination (MMSE) scores were comparable between genders. The percentage of subjects who had fallen in the previous year, number of falls/person in the previous year, and SF-8 summary scores were similar as well.

**Table 1:** Physical performance and the other measurement items.

Test	Male	Female	*p*
**Physical performance test**			
Knee extension (kg)	94.1 (40.2)	55.8 (23.3)	<0.01*
Knee flexion (kg)	50.8 (25.5)	26.8 (12.3)	<0.01*
Grip power (kg)	33.4 (8.0)	21.1 (4.8)	<0.01*
One-leg standing (sec)	34 (22)	32 (21)	0.23
Stand-up test	BLS 10 cm	BLS 10 cm	0.03*
	(1 grade up or down)	(1 grade up or down)	
Two-step score	1.40 (0.25)	1.35 (0.24)	0.03*
SMI (kg/m^2^)	7.28 (0.78)	5.99 (0.63)	<0.01*
**Cognitive function test**			
MoCA score (points)	24.7 (3.7)	25.0 (3.7)	0.41
MMSE score (points)	27.7 (2.2)	27.8 (2.0)	0.75
Experiencing fall(s) in the previous 1 year (%)	22%	20%	0.71
Number of falls in the previous 1 year/person	0.4 (0.9)	0.4 (0.8)	0.68
**HRQOL**			
SF-8 PCS (points)	47.8 (7.7)	47.6 (7.6)	0.75
SF-8 MCS (points)	50.2 (5.9)	49.4 (6.3)	0.17

*Note:* Values represent the mean (standard deviation). *Statistically significant.

*Abbreviations:* BLS, both legs standing; SMI, skeletal muscle mass index; MoCA, Montreal Cognitive Assessment; MMSE, Mini Mental State Examination; HRQOL, health-related quality of life; PCS, physical component summary; MCS, mental component summary.

Physical performance clearly affected cognitive function (Table 2). When each physical performance parameter was shifted by +1 standard deviation (SD), knee muscle strength, grip strength, one-leg standing time, and the two-step test were seen to have a significant impact on cognitive function. On the contrary, the stand-up test and Skeletal muscle mass index (SMI) were unrelated to cognitive function. Regarding age and gender covariate effects, cognitive scores became significantly lower with the negative effects of age in subjects in their 70’s and 80’s compared with those in subjects in their 50’s for all physical test analyses. No interaction between age and gender was observed. The results adjusted by gender and each physical performance parameter × gender interaction were similar to those of the former analyses. However, the estimated impacts were stronger, with the stand-up test and SMI significantly related to cognitive function.

**Table 2:** Adjusted impact of +1 SD shift of physical performance parameters on cognitive function.

Physical performance parameter	+1 SD of parameter		MoCA score	*p*	MMSE score	*p*
	Male	Female	Impact (points)		Impact (points)	
**Covariates: age group**, **gender**, **and their interaction**						
Knee extension (kg)	40.2	23.3	0.6 (0.2, 1.0)	<0.01*	0.4 (0.2, 0.6)	<0.01*
Knee flexion (kg)	25.5	12.3	0.5 (0.1, 0.9)	<0.01*	0.3 (0.1, 0.5)	<0.01*
Grip power (kg)	8.0	4.8	0.7 (0.4, 1.1)	<0.01*	0.5 (0.3, 0.8)	<0.01*
One-leg standing (sec)	22	21	0.8 (0.4, 1.2)	<0.01*	0.4 (0.2, 0.7)	<0.01*
Stand-up test (grade)	1	1	0.1 (−0.3, 0.4)	0.66	0.1 (−0.2, 0.3)	0.57
Two-step score	0.25	0.24	1.0 (0.6, 1.3)	<0.01*	0.4 (0.2, 0.7)	<0.01*
SMI (kg/m^2^)	0.78	0.63	0.1 (−0.2, 0.4)	0.43	0.1 (−0.1, 0.3)	0.30
**Covariates: gender and each physical performance parameter × gender interaction**						
Knee extension (kg)	40.2	23.3	1.4 (0.4, 2.4)	<0.01*	0.9 (0.3, 1.5)	<0.01*
Knee flexion (kg)	25.5	12.3	1.2 (0.1, 2.2)	0.02*	1.0 (0.4, 1.6)	<0.01*
Grip power (kg)	8.0	4.8	2.1 (1.1, 3.1)	<0.01*	1.2 (0.7, 1.8)	<0.01*
One-leg standing (sec)	22	21	1.8 (0.8, 2.8)	<0.01*	1.0 (0.4, 1.6)	<0.01*
Stand-up test (grade)	1	1	1.8 (0.7, 2.9)	<0.01*	0.7 (0.1, 1.3)	0.02*
Two-step score	0.25	0.24	1.7 (0.7, 2.7)	<0.01*	0.9 (0.3, 1.5)	<0.01*
SMI (kg/m^2^)	0.78	0.63	1.5 (0.4, 2.6)	<0.01*	1.1 (0.4, 1.7)	<0.01*

*Note:* Impact values represent the estimate of impact (95% confidence interval). *Statistically significant.

*Abbreviations:* SD, standard deviation; MoCA, Montreal Cognitive Assessment; MMSE, Mini Mental State Examination; SMI, skeletal muscle mass index.

Low physical performance increased the risk of falls. As seen in Table 3 summarizing the impact of each physical performance parameter on annual fall frequency, grip strength, the stand-up test, and the two-step test had significant effects on falls; a -1 SD shift increased fall risk by 39%, 23%, and 38%, respectively, from the analysis adjusted by age and gender. Although not significantly, knee extension and one-leg standing time tended to influence fall frequency. Concerning age and gender covariate effects, females in their 50’s were significantly more likely to fall than equal-age males except for the stand-up test and SMI analyses. In subjects in their 60’s and 80’s, fall frequency was significantly higher than in subjects in their 50’s for both genders. There were no significant differences for subjects in their 70’s compared with those in their 50’s. The results obtained by adjusting by gender and each physical performance parameter × gender interaction were comparable, although slightly strengthened.

**Table 3:** Adjusted impact of −1 SD shift of physical performance on annual fall frequency.

Physical performance parameter	−1 SD shift of parameter	Female	Impact on annual fall frequency	*p*
	Male		IRR	
**Covariates: age group**, **gender**, **and their interaction**				
Knee extension (kg)	−40.2	−23.3	1.2 (1.0, 1.5)	0.05
Knee flexion (kg)	−25.5	−12.3	1.0 (0.9, 1.3)	0.70
Grip power (kg)	−8.0	−4.8	1.4 (1.1, 1.7)	< 0.01*
One-leg standing (sec)	−22	−21	1.2 (1.0, 1.5)	0.05
Stand-up test (grade)	−1	−1	1.2 (1.0, 1.5)	0.04*
Two-step score	−0.25	−0.24	1.4 (1.1, 1.7)	<0.01*
SMI (kg/m^2^)	−0.78	−0.63	1.1 (0.9, 1.3)	0.20
**Covariates: gender and each physical performance parameter** × **gender interaction**				
Knee extension (kg)	−40.2	−23.3	1.3 (0.8, 2.2)	0.35
Knee flexion (kg)	−25.5	−12.3	1.1 (0.7, 1.9)	0.60
Grip power (kg)	−8.0	−4.8	1.8 (1.1, 2.9)	0.02*
One-leg standing (sec)	−22	−21	1.3 (0.8, 2.1)	0.30
Stand-up test (grade)	−1	−1	2.0 (1.2, 3.4)	0.01*
Two-step score	−0.25	−0.24	1.8 (1.2, 2.8)	<0.01*
SMI (kg/m^2^)	−0.78	−0.63	1.6 (1.0, 2.6)	0.07

*Note:* IRR represents the incidence rate ratio (95% confidence interval). *Statistically significant.

*Abbreviatifons:* SD, standard deviation; IRR, incidence rate ratio; SMI, skeletal muscle mass index.

The effect of physical performance on HRQOL was very clear (Table 4). Among SF-8 summary scores, the physical component summary (PCS) was significantly influenced by knee muscle strength, grip strength, one-leg standing time, the stand-up test, and the two-step test. The mental component summary (MCS) aspect of SF-8 did not reflect such deviations. For covariate effects, age and gender had no significant effects on the PCS. In contrast, females had significantly lower MCS scores than did males, with similar performance levels in their own gender group for knee muscle strength, grip strength, one-leg standing time, and the two-step test. Knee flexion strength, grip strength, and SMI were significantly associated with the PCS in the results adjusted by gender and each physical performance parameter × gender interaction. Lastly, we stratified the subjects into the younger group of 50–69 years and the older group of 70–89 years and performed the same regression analyses as above. The results were almost equal between the groups (data not shown).

**Table 4:** Adjusted impact of + 1 SD shift of physical performance parameters on SF-8 summary scores.

Physical performance parameter	+ 1 SD of parameter	PCS	Impact (points)	MCS	Impact (points)	*p*
	Male	Female		*p*		
**Covariates: age group**, **gender**, **and their interaction**						
Knee extension (kg)	40.2	23.3	2.0 (1.1, 2.9)	<0.01*	0.3 (−0.5, 1.1)	0.44
Knee flexion (kg)	25.5	12.3	1.7 (0.8, 2.5)	<0.01*	0.3 (−0.4, 1.0)	0.42
Grip power (kg)	8.0	4.8	1.7 (0.8, 2.6)	<0.01*	0.1 (−0.7, 0.8)	0.86
One-leg standing (sec)	22	21	1.2 (0.3, 2.2)	0.01*	0.4 (−0.4, 1.2)	0.35
Stand-up test (grade)	1	1	1.4 (0.6, 2.2)	<0.01*	0.1 (−0.6, 0.8)	0.80
Two-step score	0.25	0.24	2.0 (1.1, 2.9)	<0.01*	0.0 (−0.8, 0.8)	0.97
SMI (kg/m^2^)	0.78	0.63	0.0 (−0.7, 0.7)	0.99	0.1 (−0.5, 0.7)	0.77
**Covariates: gender and each physical performance parameter × gender interaction**						
Knee extension (kg)	40.2	23.3	1.7 (−0.5, 3.8)	0.12	−0.2 (−2.1, 1.7)	0.85
Knee flexion (kg)	25.5	12.3	2.8 (0.6, 5.0)	0.01*	0.2 (−1.6, 2.1)	0.80
Grip power (kg)	8.0	4.8	2.2 (0.0, 4.4)	0.04*	−0.5 (−2.4, 1.4)	0.59
One-leg standing (sec)	22	21	1.5 (−0.7, 3.7)	0.19	−0.3 (−2.2, 1.5)	0.73
Stand-up test (grade)	1	1	1.7 (−0.5, 4.0)	0.12	−0.6 (−2.5, 1.3)	0.52
Two-step score	0.25	0.24	1.7 (−0.5, 3.8)	0.12	−0.5 (−2.4, 1.4)	0.60
SMI (kg/m^2^)	0.78	0.63	2.7 (0.4, 5.1)	0.02*	−0.7 (−2.6, 1.1)	0.44

*Note:* Impact values represent the estimate of impact (95% confidence interval). *Statistically significant.

*Abbreviations:* SD, standard deviation; PCS, physical component summary; MCS, mental component summary; SMI, skeletal muscle mass index.

## Discussion

This study showed that in the general population of 50 to 89 years old, physical performance had a clear effect on the maintenance of HRQOL. Declining physical performance was also associated with an increase in fall risk and deterioration of cognitive function. These observations were independent of age and gender.

**Figure 1:**
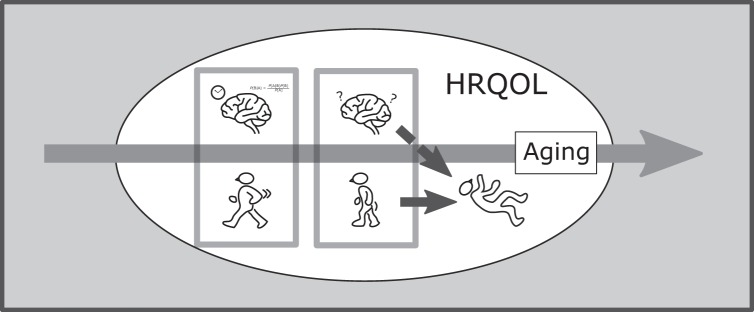
Relationship schematic among physical performance, cognitive function, falls, and HRQOL in the elderly. *Note*: Physical performance level and cognitive function fall simultaneously with age. These phenomena raise the risk of falls and represent factors lowering HRQOL. *Abbreviation:* HRQOL, health-related quality of life.

Physical performance and cognitive function were significantly related in our cohort. However, we interpreted this as the factors worsening together with age rather than exhibiting a causal relationship. The linked deterioration of physical and cognitive function tended to progress with age, leading to falls and deteriorating HRQOL (Fig. 1). Among these phenomena, perhaps the most effective interventional point for community-dwelling seniors is motor function. This model suggests that the prevention of deteriorated physical performance may facilitate HRQOL maintenance and directly or indirectly abrogate an increased need for care in older people.

Several reports have proposed that cognitive impairment leads to falls and deterioration of HRQOL. However, we did not detect any clear associations between cognitive function indicators and falls or HRQOL (data not shown). This may have been due to background differences with other studies; our cohort was sampled from the general population and therefore contained fewer cases of severely impaired cognitive function, which might have influenced HRQOL to a lesser degree.

Regarding health screening methods, the grip strength test is a useful comprehensive index of aging that is easy to perform. In past studies, grip strength was demonstrated as a comprehensive indicator of whole body muscle strength and was possibly related to bone mineral density^13–15^. Here, the relationship of grip strength with health in older people, including the aspects of cognitive function and falls, was strong. Grip strength has also been used along with decreased walking speed as a diagnostic criterion for sarcopenia^16^ and forms one of the five constituents of frailty^17^. The two-step test also showed clear correlations with cognitive function and falls. This test is part of the diagnostic criteria for locomotive syndrome by comprehensively assessing lower limb muscle strength, balance, and flexibility. As it can be carried out easily with sufficient space and a tape measure, the two-step test is applicable in various circumstances. Taken together, the grip strength test and two-step test have potential use in screening tests for cognitive deterioration and fall risk in community-dwelling older people.

This study has several limitations. First, we could not completely eliminate selection bias. Selection bias was minimized by cohort construction from random sampling of a basic resident registry. However, whether the sampled person ultimately participated in the research depended on the will of the individual, and so bias generation could not be completely avoided. Especially for older people, it was impossible for those with serious health problems to participate in some examinations, so our cohort was not considered a complete resident shrinking model. However, we produced a cohort design that was superior to a volunteer cohort design to provide evidence on the general population; in particular, the uniform age distribution in this study group minimized analysis distortion and totalization. Secondly, not all areas related to outcomes were considered. We primarily focused on physical performance and presented its impact on HRQOL. These relationships were statistically clear, but limited. There may be other factors that have a greater impact than physical performance to address HRQOL maintenance in older people. Thirdly, the frequency of falls was based on interviews and thus subject to memory bias. Lastly, this study was cross-sectional. Future longitudinal and interventional studies are currently being planned.

In the general population, diminished physical performance is related to serious problems in older citizens, specifically cognitive function deterioration, increased fall risk, and inability to maintain HRQOL. These factors are independent of age and gender, with higher physical function correlating with better status of the other conditions. The grip strength test and two-step test represent good candidates for group examinations to assess overall health in older people. The findings revealed in this study provide important evidence towards the establishment of health maintenance policies in the general population.

## Methods

This study was approved by the investigational review board of Shinshu University Hospital (approval number: 2792). This study was performed in accordance with STROBE Statement. All participants provided written informed consent for study participation. The subject on the pictures in this report provided informed consent to publish those images in an open-access, online publication.

### Construction of cohort classified by gender and age group

The participants in this study were from the Obuse study cohort, a resident cohort randomly sampled from a basic town resident registry. The cohort’s construction has been described previously^18^. The cohort included approximately 400 residents in their 50’s to 80’s whose age and gender were uniformly distributed. Table 5 shows the baseline characteristics the study population. Of the 415 participants, 412 (203 male and 209 female) were enrolled in this study, with three cases excluded for missing physical performance assessments. The majority of Obuse town residents over 50 years of age were primary or tertiary industry workers. The proportion of tertiary industry workers in their 60’s fell remarkably due to mandatory retirement, while the proportion of unemployed subjects tended to increase with age.

**Table 5:** Baseline characteristics of the study cohort.

Gender	Age	Number	Height (cm)	Weight (kg)	BMI (kg/m^2^)	Job (Pri; Sec; Ter; None)
Male	50’s	50	171.8 (6.0)	67.1 (9.1)	22.7 (2.9)	3; 7; 40; 0
	60’s	53	166.7 (4.7)	66.9 (7.7)	24.1 (2.7)	18; 5; 19; 11
	70’s	55	163.2 (5.0)	60.0 (10.3)	22.5 (3.4)	22; 2; 8; 23
	80’s	45	160.1 (5.7)	57.5 (8.5)	22.4 (2.8)	19; 0; 3; 23
	All	203	165.6 (6.8)	63.0 (9.8)	22.9 (3.0)	62; 14; 70; 57
Female	50’s	47	158.1 (4.9)	55.4 (9.0)	22.2 (3.8)	5; 4; 29; 9
	60’s	61	152.8 (5.4)	52.2 (7.6)	22.3 (2.8)	21; 4; 17; 19
	70’s	53	149.8 (5.2)	50.7 (8.0)	22.5 (3.2)	16; 3; 8; 26
	80’s	48	144.6 (5.9)	48.3 (7.9)	23.1 (3.3)	11; 0; 5; 32
	All	209	151.4 (7.1)	51.6 (8.4)	22.5 (3.3)	53; 11; 59; 86

*Note:* The table was cited from a previous study^18^. Values represent the mean (standard deviation).

*Abbreviations:* BMI, body mass index; Pri, primary industry; Sec, secondary industry; Ter, tertiary industry.

### Physical performance tests

Knee extension/flexion strength (kg) was measured with a Leg Extension/Curl and HUR Performance Recorder (HUR, Kokkola, Finland). Grip strength (kg) was determined with a hand dynamometer (Jamar Hand Dynamometer, Sammons Preston Rolyan, Bolingbrook, IL). The average values of both sides were used in evaluations. One-leg standing time was determined as the average time of both legs for each subject. Subjects who could remain standing for over 60 seconds were regarded as reaching the highest limit of the test.

**Figure 2:**
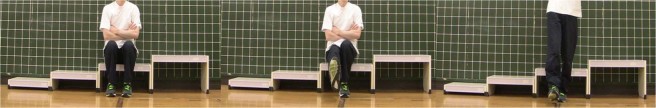
The stand-up test.

**Figure 3:**
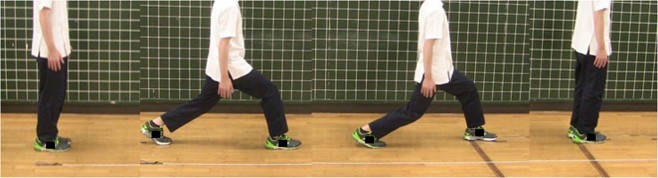
The two-step test.

The stand-up test^19^ and two-step test^20^ are physical performance tests adopted for evaluation of locomotive syndrome^21^ by the Japanese Orthopaedic Association. The stand-up test evaluates whether the subject can stand up from a sitting position on boxes of different heights (40, 30, 20, or 10 cm) with both or one leg (Fig. 2). The relative difficulty of standing from each height was as follows: 40 cm, both legs standing (BLS) < 30 cm, BLS < 20 cm, BLS < 10 cm, BLS < 40 cm, single-leg standing (SLS) < 30 cm, SLS < 20 cm, SLS < 10 cm, SLS. Successful completion of the most difficult level of standing was regarded as the final grade. For the two-step test, the subject took two maximally long strides (Fig. 3). The two-step score was determined as the length of both strides (cm)/body height (cm).

SMI was measured and calculated by dual-energy x-ray absorptiometry (Prodigy, GE Healthcare, Chicago, IL).

### Cognitive function tests

The participants of the Obuse study cohort were evaluated for cognitive function using MoCA and MMSE. As a tool to evaluate potential mild cognitive impairment (MCI), the 30-point MoCA provides higher sensitivity and specificity than does the 30-point MMSE, which is another representative cognitive function evaluation tool^22^. Both the MoCA and MMSE are interpreted as higher scores reflecting better cognitive function. For the MoCA, ≥26 points is considered normal, with possible MCI indicated by ≤25 points. Similarly for the MMSE, ≥25 points is judged as normal and ≤24 points as suspected MCI.

### HRQOL assessments

SF-8™ Health Survey measures were determined for all participants for HRQOL evaluation. Results were calculated and expressed as two summary scores: PCS and MCS.

### Statistical analyses

The mean and SD of the physical performance parameters, cognitive function scores, and SF-8 summary scores for each gender were calculated. The number of falls in the previous one year was determined via patient interviews. Differences between genders were evaluated using Welch’s t-test or Fisher’s exact test.

We examined whether physical performance impacted other important factors preserving life quality in older people, such as cognitive function and fall risk. As physical and cognitive function needed consideration for the influence of aging in each gender, we employed multivariate regression models with the value of each cognitive function test score or number of falls in the last year as the response variable, the physical performance parameters as the explanatory variable, and decade of life (ordered scale variables: 50’s, 60’s, 70’s, or 80’s), gender (male or female), and the interaction of these as the covariate. Furthermore, as another adaptation of covariate combination, gender and each physical performance parameter × gender interaction were included in regression models. Thus, the impact of physical performance on cognitive function and fall risk were estimated by multiple linear regression models and multiple Poisson regression models, respectively. SF-8 was evaluated similarly using multiple linear regression models.

Statistical analyses were carried out using the statistical package R, version 3.4.3. (available at http://www.r-project.org). The level of significance was set at *p* < 0.05.
